# Incidence and risk factors of isolated calf muscular venous thrombosis after tibial plateau fractures surgery

**DOI:** 10.1186/s12891-023-06764-5

**Published:** 2023-08-02

**Authors:** Jian Peng, Bin Feng, Weizhi Ren, Shijie Jiang, Chenying Wu, Zhenghui Hu, Wei Xu

**Affiliations:** 1 Department of Orthopedics, The Second Affiliated Hospital of Soochow University, No.1055, SanXiang Road, Gusu District, Suzhou, Jiangsu Province 215004 PR China; 2 Department of Radiology, The Second Affiliated Hospital of Soochow University, No.1055, SanXiang Road, Gusu District, Suzhou, 215004 China

**Keywords:** Tibial plateau fractures, Isolated calf muscular venous thrombosis, Incidence, Risk factors

## Abstract

**Background:**

The risks associated with deep vein thrombosis (DVT) have gained significant recognition over time. A prevalent form of distal DVT is isolated calf muscular venous thrombosis (ICMVT). Despite its common clinical occurrence, data on ICMVT subsequent to tibial plateau fracture (TPF) surgery are scarce. This study aimed to examine the epidemiological characteristics and associated risk factors (RFs) of ICMVT following TPF surgery.

**Methods:**

For this retrospective analysis, we included patients from our hospital, who underwent TPF surgery between March 2017 and March 2021. Patients’ electronic medical records were reviewed, including admission details, fracture classification, surgical procedures, and laboratory biomarkers. The HSS (The American Hospital for Special Surgery) and Rasmussen scores were employed to evaluate the clinical effect. A Color Duplex Flow Imager (CDFI) was regularly used to detect pre- and postoperative venous thrombosis in the lower limbs. Finally, uni- and multivariate logistic regression analyses were used to identify independent RFs associated with ICMVT.

**Results:**

Overall, 481 participants were recruited for analysis. Postoperative ICMVT occurred in 47 patients. All ICMVTs occurred on the affected side. Four of the 47 ICMVT patients exhibited sudden postoperative swelling in the affected limb. The HSS and Rasmussen scores in the non-ICMVT cohort (87.6 ± 8.2, 16.0 ± 1.7) were markedly different from the ICMVT cohort (84.8 ± 8.2, 15.5 ± 1.6) (*p* = 0.014, *p* = 0.031). This study finally identified five postoperative ICMVT-related RFs, which were age (> 55 years old) (OR 3.06; 95% CI 1.47–6.37; *p* = 0.003), gender (female) (OR 2.67; 95% CI 1.37–5.22; *p* = 0.004), surgical duration (> 114 min) (OR 3.14; 95% CI 1.44–6.85; *p* = 0.004), elevated white blood cell content (OR 2.85; 95% CI 1.47–5.51; *p* = 0.002), and hyponatremia (OR 2.31; 95% CI 1.04–5.12; *p* = 0.040).

**Conclusion:**

The epidemiological findings of this study may help predict ICMVT risk after surgery thus facilitating the development of individualized clinical assessments and targeted prevention programs.

## Introduction

Tibial plateau fracture (TPF) is very common in clinical practice [1]. Currently, the main treatment for obvious displacement TPF is surgery. TPFs induce a series of changes, including extremity swelling and limited mobility, leading to slow blood flow, trauma and inflammatory responses contribute to hypercoagulation, and trauma and surgical procedures can damage the vascular endothelium [2, 3]. These factors increase the risk of deep vein thrombosis (DVT) and pulmonary embolism (PE), a severe and potentially fatal complication that can occur after major orthopedic surgery. According to the location of the thrombus, DVT can be divided into proximal DVT and distal DVT. It is generally believed that proximal DVT mainly causes the occurrence of PE. However, distal DVT can also progress to proximal DVT or even PE [4–8]. Isolated calf muscular venous thrombosis (ICMVT), the most common form of distal DVT [9, 10], is more prevalent in clinical practice than proximal DVT. Reports suggest that PE is not uncommon at the initial ICMVT diagnosis, and interim follow-ups reveal that 18.8% of patients experience at least one venous thromboembolism (VTE) recurrence [11]. Other studies have reported that ICMVT is found in 23–41% of patients suspected of having DVT and in 47–79% of patients with confirmed DVT [10, 12, 13]. However, the reports on ICMVT after TPF surgery were very limited. More attention should be given to the root causes of ICMVT, and immediate intervention is necessary to prevent or delay the onset and progression of ICMVT.

In this retrospective study, we collected clinical data from patients with TPFs treated in our department to determine the incidence of ICMVT after TPFs, identify related risk factors, and evaluate their predictive capacity for ICMVT. This information could guide clinical practice and promote effective preventive strategies for this condition.

## Methods

All data for this investigation were retrieved from the TPFs database of our hospital. We retrospectively collected inpatient data from TPF patients who received surgical intervention in our hospital from March 2017 to March 2021 and conducted follow-ups via telephone questionnaires to assess patient recovery information. At present, there is no unified understanding of whether anticoagulant therapy is required for ICMVT [9, 14, 15], so we conducted Caprini scores for hospitalized patients with TPFs and only provided corresponding anticoagulant therapy for the patients with high risk before surgery [16, 17]. Eligible patients were diagnosed with ICMVT via Color Duplex Flow Imager (CDFI) on the second post-operation day and provided complementary anticoagulant therapy based on their conditions [18, 19].

### Inclusion and exclusion criteria

The following patients were included for analysis: 1. age ≥ 18 years old, undergoing TPFs surgery in our facility, and complete available hospitalization data; 2. Postoperative CDFI only revealed ICMVT. The following patients were eliminated from the analysis: 1. Pathological or old TPF (closure time from injury to surgery > three weeks); 2. Combined with other fractures requiring surgery (except fibula fractures); 3. The affected knee joint received prior surgical intervention; 4. Prior history of thrombosis and receiving anticoagulant therapy; 5. With venous thrombosis, as indicated by preoperative examination; 6. Unable to complete follow-up.

### Data collection

We queried data from the institutional electronic medical records of 615 patients who underwent TPFs surgery at our hospital between March 2017 and March 2021. Based on the inclusion and exclusion criteria, 481 patients were ultimately recruited for analysis. Based on venous thrombosis studies conducted by other researchers, we focused on the data that primarily determined ICMVT, as follows [20–22]:

1. Demographic TPFs characteristics include gender, age, body mass index (BMI), and left and right sides of the affected limb.
2. Preoperative morbidity: These included hypertension, diabetes, open fractures, and blisters, as well as a history of surgery on the affected limb.
3. Fracture classification: We used the Schatzker classification based on the comprehensive preoperative X-ray and CT three-dimensional reconstruction. These were performed by two experienced orthopedic surgeons, according to the imaging data obtained during admission. Any controversial grading was resolved through discussions among the aforementioned orthopedic surgeons. Patients were then separated into two cohorts, based on their degree of injury: Cohort 1 exhibited simple fractures (Schatzker type I-III), whereas Cohort 2 exhibited complex fractures (Schatzker type IV-VI) [23, 24].
4. Operational details: In this study, all patients were fitted with an inflatable tourniquet, set at a pressure of 40kpa, at the root of the thigh on the affected limb. The surgical duration was registered according to the operation records in the case system.
5. Laboratory indicators: The following parameters were measured upon admission: total protein, albumin, alkaline phosphatase, lactate dehydrogenase, total bilirubin, direct bilirubin, indirect bilirubin, total cholesterol, triglyceride, red blood cell count, and white blood cell count at admission. In addition, the neutrophil count, lymphocyte count, monocyte, hemoglobin level, hematocrit, platelet, fasting blood glucose, sodium concentration, potassium concentration, chlorine concentration, calcium concentration, and D -Dimer.
6. Lastly, the American Hospital for Special Surgery (HSS) score was measured during telephone-based follow-ups, and at the final outpatient visit. Imaging data were analyzed using the Rasmussen score at the last review (≥ 18 months).

### Statistical analysis

The SPSS software was employed for all data analyses. The enumeration data are given as the mean ± standard deviation (SD). The inter-group comparisons were made via the independent sample t- or Mann–Whitney U test. Categorical variables were compared using the chi-square or Fisher’s exact test and are presented as absolute values and percentages. The multivariate analysis was employed for the analysis of single factors with *p* < 0.10 or those mentioned in other studies as influencing thrombosis, and the results are presented using a binary logistic regression model as odds ratio (OR) values and 95% confidence intervals (95% CI). The Hosmer—Lemeshow (H—L) test evaluated the final model’s reasonable degree, and *p* > 0.05 was adjusted as the significance threshold.

## Results

Overall, we enrolled 481 patients, comprising 232 males (48.2%) and 249 females (51.8%), with a mean age of 51.2 ± 13.6 years (range 18–87 years). Among them, 26 (5.4%) had open fractures, and 22 (4.6%) had a preoperative blister. There were 47 cases (9.8%) of ICMVT, including 27 cases (57.4%) on the left and 20 cases (42.6%) on the right side. The population epidemiological data and single-factor analysis results are shown in Tables 1 and 2.

**Table 1:** Summary of demographic data

**Variables**	**Number (%) of ICMVT (47)**	**Number (%) of non-ICMVT (434)**	**Total**
Gender (male)	12(22.7%)	220(50.7%)	232(48.2%)
Age	58.3 ± 11.7	50.5 ± 13.6	51.2 ± 13.6
Preoperative duration (day)	5.5 ± 3.6	5.1 ± 2.7	5.5 ± 3.5
Operation time	166.9 ± 59.9	134.4 ± 58.5	137.6 ± 59.3
BMI (kg/m^2^)	24.3 ± 2.4	24.8 ± 3.0	24.8 ± 3.0
Open fractures(yes)	0(0%)	26(6%)	26(5.4%)
Blister(yes)	2(4.3%)	20(4.6%)	22(4.6%)
Hypertension(yes)	14(29.8%)	108(24.9%)	122(25.4%)
Diabetes(yes)	4(8.5%)	44(10.1%)	48(10.0%)
Fibula fracture(yes)	17(36.2%)	131(30.2%)	148(30.8%)
Left and right sides			
Left	27(57.4)	269(62%)	296(61.5%)
Right	20(42.6%)	165(38%)	185(38.5%)
Fracture type (Schatzker)			
I-III	20(42.6%)	225(51.8%)	245(50.9%)
IV-VI	27(57.4%)	209(48.2%)	236(49.1%)
WBC < lower limit	23(48.9%)	112(25.8%)	135(28.1%)
RBC < lower limit	10(21.3%)	80(18.4%)	90(18.7%)
HCT < lower limit	37(78.7%)	264(60.8%)	301(62.6%)
LYM(< 1.1 × 10^9^/L)	14(29.8%)	85(19.6%)	99(20.6%)
MON(> 0.6 × 10^9^/L)	17(36.2%)	190(43.8%)	207(43.0%)
NEUT(> 6.3 × 10^9^/L)	26(55.3%)	154(35.5%)	180(37.4%)
PLT(> 300 × 10^9^/L)	1(2.1%)	29(6.7%)	30(6.2%)
TBil(> 21 umol/L)	11(23.4%)	74(17.1%)	85(17.7%)
DBil(> 6 umol/L)	17(36.2%)	161(37.1%)	178(37.0%)
IBil(> 14 umol/L)	10(21.3%)	67(15.4%)	77(16.0%)
TC(> 5.2 mmol/L)	9(19.1%)	64(14.7%)	73(15.2%)
TG(> 1.7 mmol/L)	8(17.0%)	88(20.3%)	96(20.0%)
TP(< 60 g/L)	5(10.6%)	58(13.4%)	63(13.1%)
ALB(< 35 g/L)	4(8.5%)	28(6.5%)	32(6.7%)
ALP(> 100)	8(17.0%)	48(11.1%)	56(11.6%)
LDH(> 250 U/L)	3(6.4%)	25(5.8%)	28(5.8%)
FBG(> 6.1 mmol/L)	23(48.9%)	148(34.1%)	171 (35.6%)
K^+^(< 3.5 mmol/L)	10(21.3%)	58(13.4%)	68(14.1%)
Na^+^(< 135 mmol/L)	11(23.4%)	53(12.2%)	64(13.3%)
Ca^2+^(< 2.25 mmol/L)	27(57.4%)	250(57.6%)	277 (57.6%)
Cl^−^(< 96.0 mmol/L)	1(2.1%)	17(3.9%)	18(3.7%)
D-Dimer(> 0.50 mg/L)	47(100%)	427(98.4%)	474 (98.5%)
HBG < lower limit	2(4.3%)	39(9.0%)	41(8.5%)
HSS	84.8 ± 8.2	87.6 ± 8.2	87.3 ± 8.2
Rasmussen	15.5 ± 1.6	16.0 ± 1.7	16.0 ± 1.7

*BMI* Body mass index, *RBC* Red blood cell, reference range: females, 3.5–5.5*10^12^/L; males, 4.0–5.5*10^12^/L. *WBC* White blood cell, reference range: 4–10*10^9^/L; *HCT* Hematocrit, reference range: females, 35%-45%; males, 40%-50%. *LYM* Lymphocyte, *MON* Monocyte count, *NEUT* Neutrophile, *PLT* Platelet, 100–300*10^9^/L; *TBIL* Total bilirubin, *DBIL* Direct bilirubin, *IBIL* Indirect bilirubin, *TC* Total cholesterol, *TG* Triglyceride, *TP* Total protein, *ALB* Albumin, *ALP* Alkaline phosphatase, *LDH* Lactate dehydrogenase, *FBG* Fasting blood glucose, *HGB* Hemoglobin, reference range: females, 110–150 g/L; males, 120–160 g/L, *Na*^*+*^ Sodium concentration, *K*^*+*^ Potassium concentration, *Cl*^*−*^ Chlorine concentration, *Ca*^*2+*^ Calcium concentration. *HSS* The American Hospital for Special Surgery

**Table 2:** The ICMVT-based risk factor after tibial plateau fractures surgery according to univariate analysis

**Variables**	**Number (%) of ICMVT (47)**	**Number (%) of non-ICMVT (434)**	***P***
Gender (male)	12(22.7%)	220(50.7%)	0.001
Age	58.3 ± 11.7	50.5 ± 13.6	< 0.001
> 55 years old	30(63.8%)	166(38.2%)	
Operation time	166.9 ± 59.9	134.4 ± 58.5	< 0.001
> 114 min	38(80.9%)	237(54.6%)	
Open fractures(yes)	0(0%)	26(6%)	0.096
WBC < lower limit	23(48.9%)	112(25.8%)	< 0.001
HCT < lower limit	37(78.7%)	264(60.8%)	0.016
NEUT(> 6.3 × 10^9^/L)	26(55.3%)	154(35.5%)	0.008
FBG(> 6.1 mmol/L)	23(48.9%)	148(34.1%)	0.044
Na^+^(< 135 mmol/L)	11(23.4%)	53(12.2%)	0.032
D-Dimer(> 0.50 mg/L)	47(100%)	427(98.4%)	1.000
HSS	84.8 ± 8.2	87.6 ± 8.2	0.014
Rasmussen	15.5 ± 1.6	16.0 ± 1.7	0.031

**Fig. 1:**
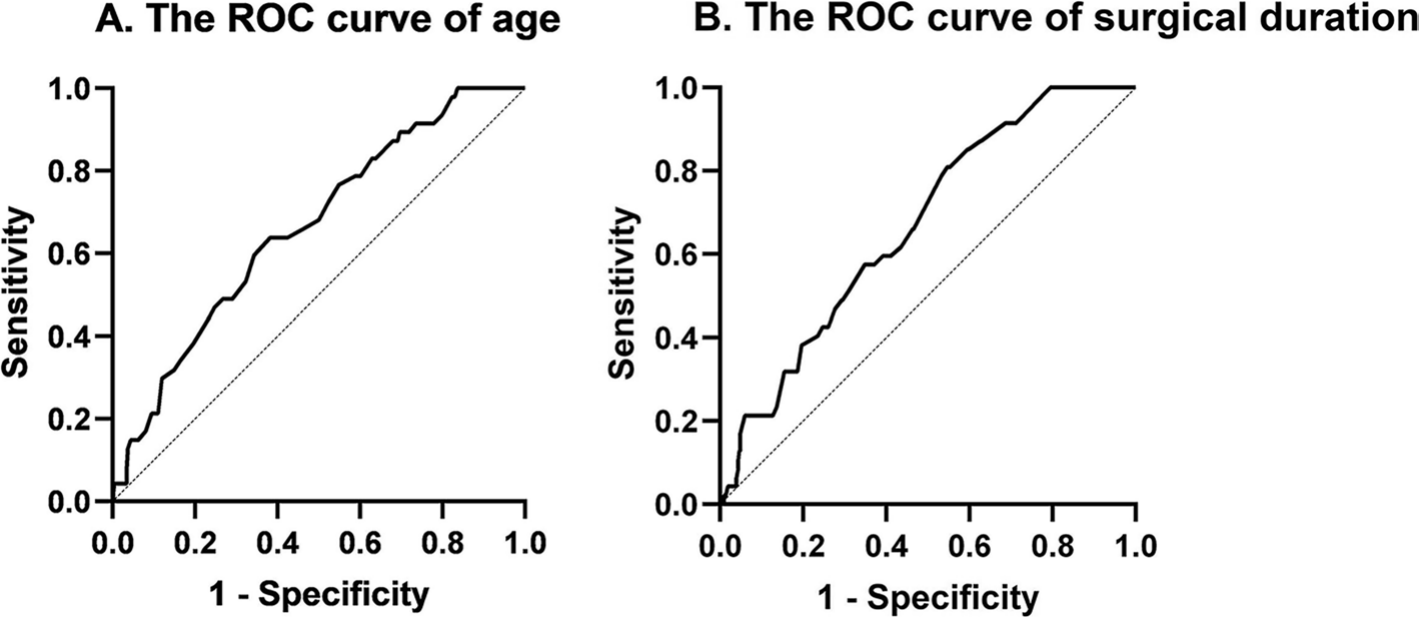
**A** The ROC curve analysis revealed that, at a cutoff point of 55.5 years old, the sensitivity and specificity of age in predicting ICMVT were 0.64 and 0.62, respectively, and the area under the curve (AUC) was 0.66. **B** At a cutoff point of 114.5 min, the sensitivity and specificity of surgical duration in predicting ICMVT were 0.81 and 0.45, respectively, and the AUC was 0.67

The age and surgical duration of the ICMVT cohort were markedly elevated compared to the non-ICMVT cohort (*p* < 0.001). Given the impact of patient age and surgical duration on ICMVT, we generated receiver operating characteristic (ROC) curves to determine the optimal threshold values for age and surgical duration to predict ICMVT. In addition, we employed area under curve (AUC) analysis to assess the significance of the ROC curve (Fig. 1a, b). As shown in Table 1, significant differences were observed between the ICMVT and non-ICMVT cohorts regarding gender (*p* = 0.001), age (*p* < 0.001), surgical duration (*p* < 0.001), white blood cell (WBC) count (*p* < 0.001), elevated neutrophils levels (*p* = 0.008), hyponatremia (*p* = 0.032), fasting blood glucose (*p* = 0.044), and hematocrit levels (*p* = 0.016). Next, the baseline variables that reached significance in univariate analysis were entered into multivariate analysis. The eligible variables were carefully selected, given the number of available events, to ensure the parsimony of the final model [20, 25–30]. Finally, the D-Dimer level (*p* = 1.000), the presence of an open fracture (*p* = 0.096), and the previously mentioned eight variables were incorporated into the binary logistic regression model (Table 3).

**Table 3:** Multivariate assessment of ICMVT-related factors after tibial plateau fractures

**Variables**	**OR**	**95%CI**	***P***
Age(> 55 years old)	3.06	1.47–6.37	0.003
Gender(female)	2.67	1.37–5.22	0.004
Surgical duration(> 114 min)	3.14	1.44–6.85	0.004
WBC < lower limit	2.85	1.47–5.51	0.002
Na^+^(< 135 mmol/L)	2.31	1.04–5.12	0.040

Numbers are significant at *p* < 0.05, *OR* Odds ratio, *CI* Confidence interval. *WBC* White blood cell, reference range: 4–10*109/L. (Open fracture, *p* = 0.60; *NEUT* Neutrophile, *p* = 0.079; D-Dimer, *p* = 0.545; *HCT* Hematocrit, *p* = 0.189)

We identified five ICMVT-related RFs in the final analysis: age (> 55 years old) (OR 3.06; 95% CI 1.47–6.37; *p* = 0.003), gender (female) (OR 2.67; 95% CI 1.37–5.22; *p* = 0.004), surgical duration (> 114 min) (OR 3.14; 95% CI 1.44–6.85; *p* = 0.004), elevated WBC count (OR 2.85; 95% CI 1.47–5.51; *p* = 0.002), and hyponatremia (OR 2.31; 95% CI 1.04–5.12; *p* = 0.040). Moreover, the H–L test revealed that the final model displayed an elevated degree of fit (*X*^2^ = 5.03, *p* = 0.75; Nagelkerke *R*^2^ = 0.20).

## Discussion

This study revealed that the patient age (> 55 years old), gender (female), surgical duration (> 114 min), elevated WBC levels, and hyponatremia (< 135 mmol/L) were independent RFs for postoperative ICMVT occurrence in patients with TPFs. The HSS and Rasmussen scores of patients with ICMVT were significantly lower than those without ICMVT (*p* = 0.014, *p* = 0.031).

The displacement fracture of the tibial plateau and operation may lead to popliteal vascular injury, an important factor for the formation of popliteal vein thrombus, a form of proximal DVT. With increasing awareness of the serious hazards of DVT, various measures have been taken for DVT prophylaxis, such as low molecular weight heparin, graduated compression stockings, and intermittent pneumatic compression devices. The incidence of proximal DVT has dropped significantly. In the past four years, only four patients with TPFs developed proximal DVT in our hospital, and the incidence was 0.83%. In contrast, the incidence of postoperative ICMVT was 9.8%, which is considerably higher than proximal DVT, a conclusion supported by previous research [10–13]. The occurrence of ICMVT is more common in clinical practice, and due to the possibility of further progress, further analysis of its epidemiological data is needed. Therefore, we mainly analyzed patients with postoperative ICMVT. Four of the 47 patients diagnosed with postoperative ICMVT exhibited sudden swelling of the affected limb. Based on the CDFI, ICMVT was suggested. The symptoms of 4 patients were alleviated upon anticoagulation adjustment, and no thrombosis was evident one month after surgery. All ICMVTs occurred on the affected side, which may result from the vascular endothelial injury caused by the trauma and surgery on the affected side, as well as the systemic hypercoagulation state and extremity swelling due to fracture surgery.

This study considered age (*p* = 0.003) as an independent RF for ICMVT. Based on the ROC curve analysis for age, the optimal threshold was 55.5 years old. It carried a sensitivity of 0.64 and specificity of 0.62, thus, indicating the enhanced predictability of age in predicting ICMVT after TPFs surgery. Similar results were confirmed in the study of Li et al. [21]. The average age of patients in their study was 45.2 years old, compared with 51.2 years old in ours, which makes our threshold higher than other studies have found. This may potentially be due to the alterations in blood components that occur with age, which may, in turn, favor thrombus clotting. This suggests that ICMVT may be more common in older patients with fractures [31].

Prolonged surgical duration (*p* = 0.004) was also found to be correlated with postoperative ICMVT development in this study. The optimal threshold, as evidenced by the ROC curve, was 114.5 min. Above this critical value, the postoperative ICMVT risk increased. It is well established that thrombus formation requires three factors: venous blood flow stasis, vascular endothelial injury, and blood hypercoagulability [32, 33]. Due to the application of a tourniquet, blood stasis can occur within the veins, thereby decreasing blood flow velocity during surgery. Meanwhile, the operation is a form of trauma. The trauma- and anesthesia-associated stress and inflammatory response often damage the vascular endothelium. Studies involving routine emergency surgical procedures reported that prolonged surgical duration is closely related to VTE [34]. Li et al. also revealed that DVT formation after TPF is correlated with the prolongation of operation duration [21]. In this study, the ROC curve was used to determine the critical value of operation time, beyond which the probability of postoperative thrombosis increased, which was the same as the conclusion of previous studies. Therefore, we concluded that a prolonged surgical duration might be one of the RFs for lower extremity ICMVT.

Along with demographic indicators, we also demonstrated an association between hyponatremia, elevated WBC count, and postoperative ICMVT. Several studies reported that hyponatremia impacts venous thrombosis before and after TPFs, in which the cause of hyponatremia was not well elucidated. Based on reports in other relevant literature, we speculated that hyponatremia might be related to the patient’s morbidity and inflammatory response. The study showed patient morbidities, such as cardiac or renal insufficiency and alteration of blood flow status, accompanied by hyponatremia and patients with hyponatremia had a higher rate of new fractures [35]. Hyponatremia was reported to be significantly correlated with inflammatory response [36]. Previous studies reported that inflammatory responses were related to thrombosis formation [22, 28, 37–39]. In addition, Liu et al. concluded that an enhanced neutrophil content was significantly associated with venous thrombosis formation after TPFs [28]. However, our study results did not reflect this conclusion (*p* = 0.079). In this investigation, elevated WBC levels were strongly associated with ICMVT after TPFs (*p* = 0.002). WBC is prominent participants in inflammatory development and is closely related to whole body inflammation [40]. On the other hand, WBC primarily took part in forming the front edge of the thrombus, and they promote further thrombosis development using tissue factors [41]. Therefore, an increase in WBC after a tibial plateau fracture may accelerate thrombus formation.

In our findings, the HSS scores (87.6 ± 8.2) and Rasmussen scores (16.0 ± 1.7) of patients without ICMVT were significantly different from those of patients with ICMVT (84.8 ± 8.2, 15.5 ± 1.6) (*p* = 0.014, *p* = 0.031). Several earlier reports suggested that the HSS score was strongly associated with TPFs classification [42]. However, concerning the ICMVT incidence, we found no significant difference between complex and simple fractures based on the Schatzker stratification (*p* = 0.226). Other studies also showed that fracture classification of the tibial plateau didn’t affect thrombus formation [21]. Based on our results, the prognosis of patients with TPFs without ICMVT was better than the TPFs patients with ICMVT, thereby suggesting that ICMVT is a risk factor affecting patient prognosis. On the other hand, the Rasmussen imaging score is often related to fracture severity. Moreover, the Rasmussen score of patients with complex fractures is generally markedly lower than patients with simple fractures [43, 44]. In this investigation, the Rasmussen score was significantly distinct between with and without ICMVT cohorts. This evidence suggests that with more severe fractures, the surgical duration tends to be more prolonged, simultaneously increasing the incidence of ICMVT. However, there seems to be some contradiction, so further studies are required to verify the predictability of postoperative recovery in patients with TPFs.

Previous studies on DVT mainly focus on the development of proximal DVT, including the direction of hip fracture, pelvic fracture, and hip and knee replacement. This study focuses on the analysis of distal DVT, which has a higher incidence following postoperative tibial plateau fractures, intending to personalize patient assessment and develop targeted prevention programs within the clinic.

This investigation had certain limitations. Firstly, there was no consensus on the necessity of anticoagulants, and the thrombosis prevention approach after TPFs. Secondly, the impact of postoperative routine anticoagulation administration on ICMVT formation was not considered. Despite these limitations, the findings are useful for personalizing patient assessment and in developing targeted prevention programs within the clinic. Future studies will further explore the effect of prophylactic anticoagulation administration on the occurrence of ICMVT and the postoperative outcomes of TPFs.

## Conclusion

This investigation revealed a 9.8% incidence of postoperative ICMVT in patients with TPFs, all occurring on the affected side. The HSS and Rasmussen scores in the non-ICMVT cohort were markedly enhanced, compared to the ICMVT cohort, indicating that the ICMVT may affect fracture prognosis. We identified five RFs: age, gender, prolonged surgical duration, elevated WBC levels, and hyponatremia.

## Data Availability

The authors confirm that the data supporting the findings of this study are available within the article.

## Abbreviations

DVT: Deep vein thrombosis
ICMVT: Isolated calf muscular venous thrombosis
TPFs: Tibial plateau fractures
RFs: Risk factors
HSS: The American Hospital for Special Surgery
CDFI: Color Duplex Flow Imager
PE: Pulmonary embolism
VTE: Venous thromboembolism
OR: Odds ratio
CI: Confidence intervals
H—L: Hosmer – Lemeshow
ROC: Receiver operating characteristic
AUC: Area under curve
